# Effect of herbal extracts on peripheral nerve regeneration after microsurgery of the sciatic nerve in rats

**DOI:** 10.1186/s12906-021-03335-w

**Published:** 2021-06-04

**Authors:** Young Jun Kim, Kyu Jin Kim, Jae Hoon Lee, Seong-Uk Park, Seung-Yeon Cho

**Affiliations:** 1 Department of Orthopedic Surgery, Kyung Hee University Hospital at Gangdong, School of Medicine, Kyung Hee University, 892 Dongnam-ro, Gangdong-gu, Seoul, Republic of Korea; 2 Department of Cardiology and Neurology, Kyung Hee University Hospital at Gangdong, College of Korean Medicine, Kyung Hee University, 892 Dongnam-ro, Gangdong-gu, Seoul, Republic of Korea; 3 Department of Cardiology and Neurology, College of Korean Medicine, Kyung Hee University, 26 Kyungheedae-ro, Dongdaemun-gu, Seoul, Republic of Korea

**Keywords:** Herbal medicine, Microsurgery, Nerve regeneration, Peripheral nerve injury

## Abstract

**Background:**

Recent experimental studies using herbal extracts have shown the possibility of peripheral nerve regeneration. This study aimed to investigate the effects of herbal extracts on peripheral nerve regeneration in a rat sciatic nerve injury model.

**Methods:**

A total of 53 rats were randomly assigned to a control group or one of four experimental groups. In all rats, the sciatic nerve was completely severed and microscopic epineural end-to-end neurorrhaphy was performed. Normal saline (2 mL) was topically applied to the site of nerve repair in the control group, whereas four different herbal extracts – 2 mL each of *Astragalus mongholicus* Bunge, *Coptis japonica* (Thunb.) Makino, *Aconitum carmichaelii* Debeaux, *or Paeonia lactiflora* Pall. – were topically applied to the site of nerve repair in each experimental group. Nerve conduction studies were performed at an average of 11.9 weeks after the operation, and conduction velocity and proximal and distal amplitudes were measured. Biopsies were performed at an average of 13.2 weeks after the initial neurorrhaphy. The quality of nerve anastomosis and perineural adhesion to the surrounding soft tissues was macroscopically evaluated. The neuroma size at the site of the neurorrhaphy was microscopically measured, whereas the size of the scar tissue was evaluated relative to the diameter of the repaired nerve.

**Results:**

The nerve conduction study results showed the highest nerve conduction velocity in the experimental group that used the *Coptis japonica* (Thunb.) Makino extract and the highest proximal and distal amplitudes in the experimental group that used the *Aconitum carmichaelii* Debeaux extract. Macroscopic evaluations after the second operation showed that grade 2 perineural adhesion was found in 70.8% of rats. The mean neuroma size in the *Coptis japonica* (Thunb.) Makino, *Aconitum carmichaelii* Debeaux, and *Paeonia lactiflora* Pall. groups showed statistically significant decreases relative to the control group. The mean scar tissue formation index in the *Paeonia lactiflora* Pall. group showed a statistically significant decrease relative to the control group.

**Conclusions:**

The peripheral nerve regeneration effect of the herbal extracts was confirmed through decreased neuroma and scar tissue formation.

**Supplementary Information:**

The online version contains supplementary material available at 10.1186/s12906-021-03335-w.

## Background

Peripheral nerve injury is often seen clinically, and when nerve injury is caused by a sharp knife, glass laceration, or fracture of the diaphysis, the nerve may get completely cut. These cases account for approximately 30% of severe nerve injuries [1]. Before the eighteenth century, it was known that peripheral nerve regeneration did not occur. However, many studies on peripheral nerves have been conducted since then, and peripheral nerve regeneration is now understood to be a complicated process that involves various factors [2].

Neurotmesis, the most severe type of peripheral nerve injury according to Seddon’s classification, is the complete cutting of the nerve due to laceration or severe trauma [3]. It refers to the condition in which the continuity of the nerve trunk is lost, and the endoneurium, perineurium, and epineurium (the supporting tissues around the axon) are ruptured. Therefore, spontaneous regeneration of the nerve does not occur, and the prognosis is poor if connectivity is not recovered via surgical methods such as neurorrhaphy or nerve graft which uses a microsurgical technique.

However, while microsurgery may regenerate fascicular groups or individual fascicles, they are unable to regenerate nerves (i.e., the regeneration of individual axons at the neurophysiological molecular level). Due to this limitation, nerve regeneration is often incomplete and sometimes has a poor prognosis. Therefore, various studies have been performed to overcome this problem. These methods are broadly classified into those that (a) promote axon regeneration and those that (b) reduce the inflammatory reaction of the surrounding environment that causes scar tissue formation [4].

To date, there have been no clinically applicable drugs, leaving surgery as the only option, but various materials have been studied to promote axonal sprouting and improve nerve regeneration. Several studies have used neurotrophic factors and reported the effects of several factors on stumps after nerve injury. Although nerve growth factors showed the most successful result [5, 6], there has been no report that it is superior to nerve autotransplantation in nerve defect models [4, 7]. There are concerns about the treatment period, dosage, side effects, and unexpected interactions between various nerve growth factors [8].

Scar tissue formation after nerve injury interferes with axon regeneration, is inversely proportional to functional recovery, and is associated with a decreased diameter of regenerated nerves, decreased herniated nerve fibers, and the formation of neuromas [9, 10]. There are no clinically recommended drugs to decrease scar tissue; therefore, corticosteroids have been used the most.

Several herbal medicines have been known to be effective in recovering from nerve injury. They have shown a recovery effect on the central nervous system as well as on peripheral nerves. Several herbal medicines have multifactorial actions and fewer side effects than conventional drugs; therefore, several studies have been carried out using medicinal herbs [11]. Most of the existing animal studies have used a rat model of sciatic nerve crush injury to confirm the nerve regeneration effect of herbal medicines [12–14]. However, there is a limit in estimating the precise effect of the herbs on nerve regeneration because this model causes imperfect nerve injury through clamps, thereby making spontaneous nerve regeneration possible. Moreover, existing studies on herbal medicines have involved peritoneal injection or oral administration of herbal extracts, which may be affected by enzymatic degradation. In addition, systematic adverse effects may rarely occur [14]. In particular, it is not known whether herbal medicines are effective for nerve regeneration when the nerve is cut and operated. Therefore, in this study, we investigated the direct effect of herbs on nerve regeneration and scar tissue formation by topical application of herbal extracts after nerve microsurgery using a sciatic nerve transection model.

## Methods

### Ethics approval

This study was performed according to international, national and institutional rules considering animal experiments. The protocol was approved by the Institutional Animal Care and Use Committee (IACUC) of Kyung Hee University Hospital at Gangdong (KHNMC AP 2015–007).

### Experimental animals

Fifty-three adult male 8–10-week-old Sprague-Dawley rats, each weighing 300–400 g, were purchased from KOATECH (Gyeonggi-do, Republic of Korea). In a standardized animal laboratory where temperature, humidity, and illumination were kept constant, the animals were allowed to adapt to the environment for 2 weeks. They were housed (two per cage) and fed food and water ad libitum. The rats were randomly assigned to a control group or one of the four experimental groups using a computer-based random order generator. The control group consisted of 13 rats, and each of the four experimental groups consisted of 10 rats. This preliminary study was conducted with a small sample size, according to the recommendations of the IACUC. At the end of the study, the animals were sacrificed by exposure to gradually increasing concentrations of carbon oxide (CO_2_) in an enclosed container until complete cessation of breathing was observed for a minimum of 2 min.

### Herbal drug extraction

Herbal drug pharmacists at the hospital manufactured the experimental drugs. The herbal drugs used were *Astragalus mongholicus* Bunge*, Coptis japonica* (Thunb.) Makino, *Aconitum carmichaelii* Debeaux, and *Paeonia lactiflora* Pall. For each herb, an 80 g sample was washed, sliced, and placed in a round flask with 800 mL of distilled water. The experimental flask was then placed in a heating mantle and connected to a condenser. The mixture was heated at 100 °C for 2 h and filtered through a syringe filter with a diameter of 0.22 μL. Individual aliquots (20 mL) of extract were placed in vials, covered with a rubber stopper and aluminum cap, and sterilized in a high-pressure steam sterilizer at 121 °C for 30 min. After cooling to room temperature, the vials were stored in a drug freezer until use and discarded 2 weeks after opening. Each herbal extract was analyzed using liquid chromatography coupled with mass spectrometry (See Additional file 1 and Supplementary Figure 1, 2, 3 and 4).

### Surgery method

Surgery was performed on the left sciatic nerve in all experimental subjects. Anesthesia was performed by mixing 0.7 mg/kg of Zoletil™ 250 mg/5 cc (Virbac, Tiletamine + Zolazepam, 1:1) with 0.14 mL of Rompun™ 0.02 mL/100 g (Bayer, Xylazine) and applied via intraperitoneal injection. After anesthesia, the left gluteal region and thigh were shaved, disinfected with betadine solution, and fixed on the surgical plate. The nerves surrounded by the fascia were then exposed using the gluteal splitting approach. A surgical microscope (Carl Zeiss f170) and microsurgery instrument set were used. The soft tissue was carefully removed to confirm the path of the sciatic nerve. The middle of the exposed sciatic nerve was finely cut with microsurgical scissors, and a 10–0 monofilament polyamide nylon suture (Ethicon; Johnson & Johnson, Somerville, NJ, USA) was used to perform epineural end-to-end neurorrhaphy. In the control group, 2 mL of normal saline was applied, and in each experimental group, 2 mL of herb extract was topically applied between the femoral muscles that underwent longitudinal splitting at the site of neurorrhaphy. The herbal extract or normal saline was administered once during neurorrhaphy. After confirming that the 2 mL of the experimental drug was absorbed, 2 mL was administered.

The cut femoral muscle was then sutured two to three times using a 4–0 monofilament nylon suture to reduce leakage of the experimental drug. After recovery from anesthesia, the animals were not restricted in activity. The skin suture site was kept exposed, and no disinfection or removal of the thread was performed. The surgeon was the only person aware of the treatment group allocation.

### Evaluation method

#### Nerve conduction study

All evaluation was performed by investigators blinded to treatment allocation. Experimental animals at an average age of 11.9 weeks (range; 9.1–13.7) were anesthetized prior to the nerve conduction study with the same dose used during surgery. Thereafter, the conduction velocity and amplitude of compound muscle action potentials were recorded. The compound muscle action potentials were recorded by inserting a needle-shaped recording electrode into the motion point of the plantar muscle and finding the position where the first negative phase potential of the greatest amplitude was recorded. The stimulating electrode examined the sciatic nerve by stimulating points 2 cm proximal and 3 cm distal from the recorded position. The conduction velocity was calculated as the distance between the proximal and distal stimulation points (10 mm) divided by the latency difference between the proximal and distal parts. The amplitude was calculated as the peak-to-peak amplitude. Electromyography was performed using Synergy® (Oxford Medelec, Wiesbaden, Germany), and the frequency range was 10–1000 kHz. Sensitivity and sweep speed were adjusted to 5–10 mV/division and 50 m/s/division, respectively, as necessary.

#### Macroscopic evaluation

After an average of 13.2 weeks (range; 12.0–15.1), lethal doses of anesthetics were administered, and all subjects were killed and sent for re-surgery. The previously described procedure was used to expose the previous surgical site. After confirming the quality of nerve anastomosis and perineural adhesion at the site of the 10–0 monofilament nylon suture, a biopsy of the entire sciatic nerve and perineural tissue was performed. Cases with no connectivity at the neurorrhaphy site or a thin site that was not considered as nerve tissue were regarded as failures. Perineural adhesion was evaluated using a numerical grading system described by Petersen et al. [15]. The degree of healing of the surrounding skin and muscle fascia and the degree of nerve adherence between the nerve and surrounding tissue were evaluated (Table 1).

**Table 1:** Numerical grading system for gross evaluation, as proposed by Peterson et al. [15]

Tissue	Grade	Definition
Skin and muscle fascia	1	Entirely closed
	2	Partially open
	3	Completely open
Nerve adherence	1	No dissection or mild blunt dissection
	2	Some vigorous blunt dissection
	3	Sharp dissection required

#### Microscopic evaluation

**Fig. 1:**
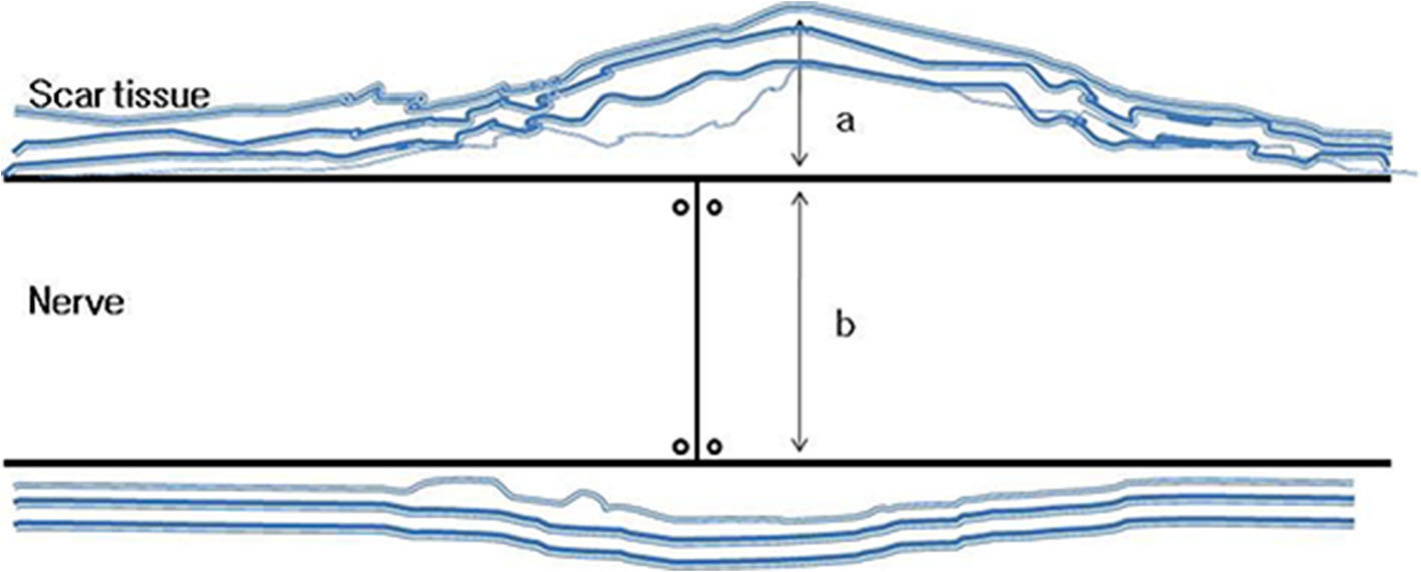
Schematic diagram of the scar tissue formation index. The scar tissue with at the thickest point was normalized by dividing it by the nerve diameter. Scar tissue formation index = ratio of the thickness of the epineural scar tissue to the nerve diameter (a/b)

The collected tissues were fixed in 10% neutral formalin and embedded in paraffin. Tissue slices were made in the longitudinal direction of the nerve tissue with a thickness of 5 μm, and hematoxylin-eosin (H-E) staining was performed to prepare the samples. The samples were evaluated by a blinded pathologist using an optical microscope at 40× magnification. Neuroma size was measured by comparing the diameter of the nerve at the site of neurorrhaphy to that on the normal proximal nerve in each group. If the diameter of the measured nerve was greater than double that of the normal nerve, then it was recorded as 2. The thickness of the thickest part of the scar tissue around the neurorrhaphy site was measured and compared with the diameter of the sutured nerve to evaluate the scar tissue formation index [16]. The thickness of the soft tissue was recorded as 0.5 when the thickness of the soft tissue was half of the thickness of the nerve (Fig. 1).

### Statistical analysis

Statistical analysis was performed using IBM SPSS Statistics 20.0 (IBM Corp., New York). Nerve conduction study results were analyzed via one-way ANOVA. For the macroscopic evaluation, the degree of adhesion of the control and experimental groups was analyzed using a linear by linear association test. For the microscopic evaluation, a Kruskal-Wallis test was performed. The post-hoc analysis set up a *P* value of less than 0.0125 as significant for microscopic evaluation and a P value of less than 0.05 for other cases.

## Results

**Fig. 2:**
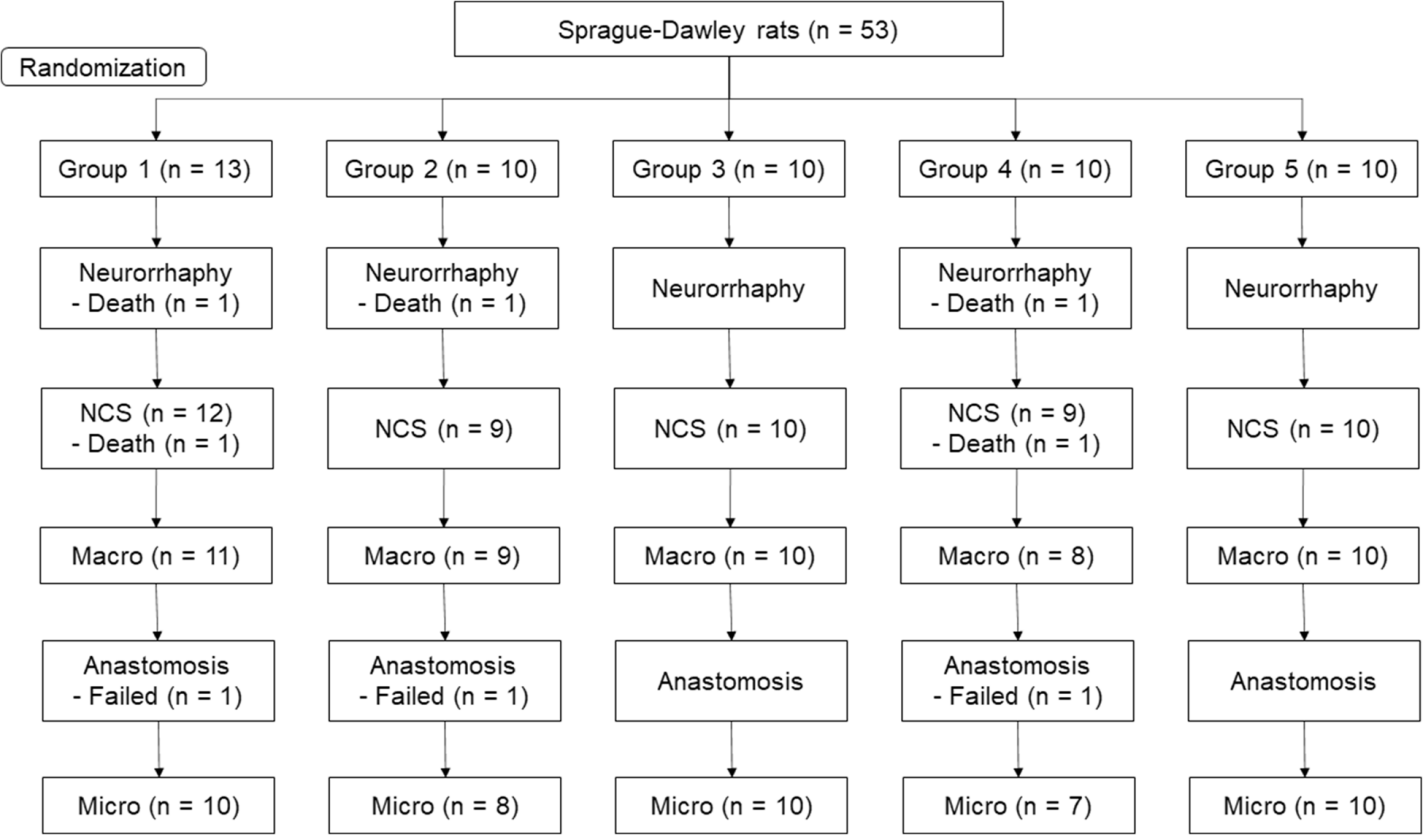
Flow chart of the animal experiments. Number of rats included or excluded in each experiment. Group 1: Control, Group 2: *Astragalus mongholicus* Bunge, Group 3: *Coptis japonica* (Thunb.) Makino, Group 4: *Aconitum carmichaelii* Debeaux, Group 5: *Paeonia lactiflora* Pall. NCS: nerve conduction study, Macro: macroscopic evaluation, Micro: microscopic evaluation

Three rats died after neurorrhaphy, and the remaining 50 rats underwent the nerve conduction study. After the nerve conduction study, two rats died prior to re-operation, and 48 rats underwent re-operation. With the exception of three rats in which the surgical anastomosis failed, as observed in the macroscopic evaluation during re-operation, 45 rats underwent microscopic evaluation. Analysis of the nerve conduction study was performed in 47 rats, excluding the three rats in which anastomosis failed (Fig. 2).

**Fig. 3:**
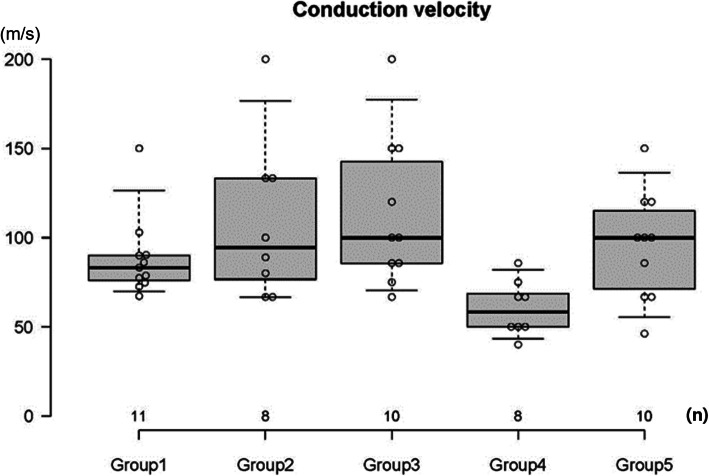
Results of the nerve conduction velocity for each group. There was no statistically significant difference from the control group. Group 1: Control, Group 2: *Astragalus mongholicus* Bunge, Group 3: *Coptis japonica* (Thunb.) Makino, Group 4: *Aconitum carmichaelii* Debeaux, Group 5: *Paeonia lactiflora* Pall

**Fig. 4:**
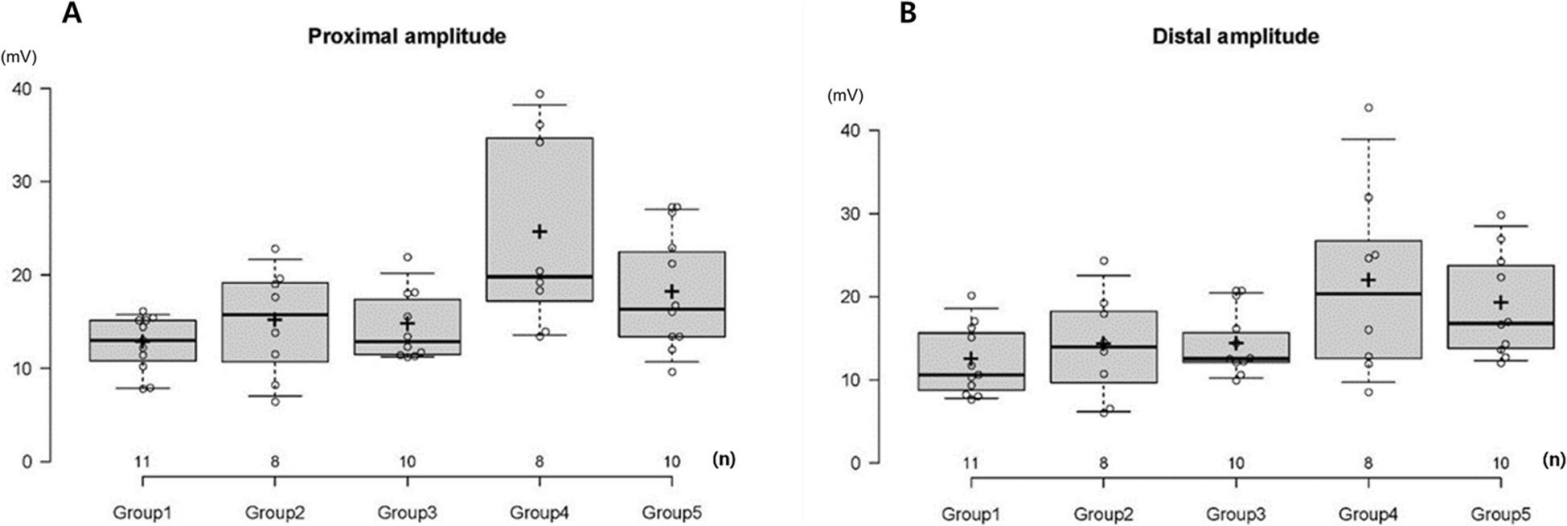
Results of the proximal and distal amplitude in each group. (**A**) Proximal amplitude (**B**) Distal amplitude. Group 1: Control, Group 2: *Astragalus mongholicus* Bunge, Group 3: *Coptis japonica* (Thunb.) Makino, Group 4: *Aconitum carmichaelii* Debeaux, Group 5: *Paeonia lactiflora* Pall. Only the *Aconitum carmichaelii* Debeaux group had significantly higher proximal and distal amplitudes than the control group (*p* = 0.002 and *p* = 0.041, respectively)

Nerve conduction studies were performed on 50 rats, except the three rats that died before the study. The analysis was conducted on 47 subjects, after excluding the three subjects in whom anastomosis failed. Nerve conduction velocity was an average of 88.45 m/s in the control group. It was the highest in the *Coptis japonica* (Thunb.) Makino group, with an average of 113.31 m/s. The *Astragalus mongholicus* Bunge and *Paeonia lactiflora* Pall. groups showed average values of 108.61 m/s and 95.53 m/s, respectively. These values were higher than those in the control group, but the difference was not statistically significant. The *Aconitum carmichaelii* Debeaux group had a lower result than the control group, with an average velocity of 60.51 m/s, but the difference was not statistically significant. The amplitude for the proximal part was measured in the control group, with an average of 12.60 mV; it was higher in all experimental groups than in the control group. The *Aconitum carmichaelii* Debeaux group had the highest value, with an average of 24.36 mV, and the difference was statistically significant (*p* = 0.002). The average amplitude of the distal part was 12.19 mV in the control group, and all the experimental groups showed increased values compared to the control group. Regarding the distal amplitude, the *Aconitum carmichaelii* Debeaux group had the highest value, with an average of 21.68 mV, and the difference was statistically significant (*p* = 0.041). The other groups were not statistically different from the control group based on the proximal or distal amplitudes (Table 2, Fig. 3–Fig. 4).

**Table 2:** Results of the nerve conduction study in each group

Group (n)	NCV (m/s)	PA (mV)	DA (mV)
Group 1 (11)	88.45 ± 22.68	12.60 ± 2.97	12.19 ± 4.24
Group 2 (8)	108.61 ± 45.30	14.86 ± 5.84	14.06 ± 6.33
Group 3 (10)	113.31 ± 41.86	14.48 ± 3.74	14.12 ± 3.74
Group 4 (8)	60.51 ± 15.46	24.36 ± 10.48^a^	21.68 ± 11.63^a^
Group 5 (10)	95.53 ± 30.58	17.92 ± 6.24	18.93 ± 6.39

Group 1: Control, Group 2: *Astragalus mongholicus* Bunge, Group 3: *Coptis japonica* (Thunb.) Makino, Group 4: *Aconitum carmichaelii* Debeaux, Group 5: *Paeonia lactiflora* Pall

*NCV* Nerve conduction velocity, *PA* Proximal amplitude, *DA* Distal amplitude

^a^One-way ANOVA

Only the *Aconitum carmichaelii* Debeaux group had a significantly higher amplitude in the proximal and distal parts than the control group (*p* = 0.002 and *p* = 0.041, respectively)

**Fig. 5:**
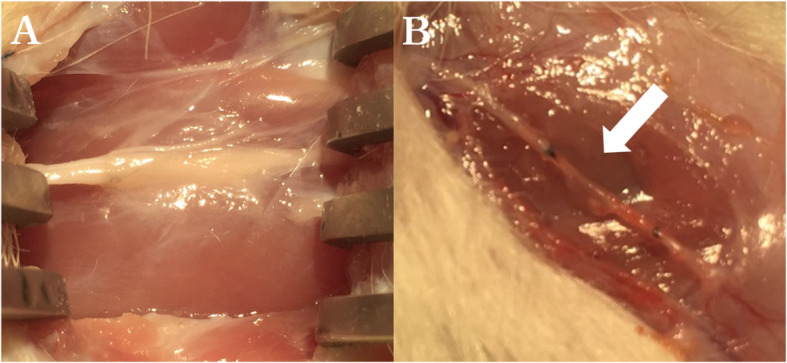
Surgical microscopy images showing subjects with excellent connectivity (**A**) and anastomosis failure (**B**). The anastomosis area possessed reduced thickness and became transparent tissue (white arrow)

During re-operation, in three subjects, the neurorrhaphy site was significantly thinner and more transparent than the proximal and distal parts even when observed with the naked eye; therefore, it was determined that the neurorrhaphy failed (Fig. 5). These three subjects were excluded from the nerve conduction study and microscopic evaluation (Fig. 2). The results of the 48 subjects, according to the Peterson classification [15], showed a grade 1 degree of skin and fascia healing in all cases, thereby showing that no problems existed in wound healing at the surgical site. Grade 1 perineural adhesion was observed in three subjects (6.3%), grade 2 was observed in 34 subjects (70.8%), and grade 3 was observed in 11 subjects (22.9%). In most cases, there was a moderate degree of adhesion between the surgical site and the anastomotic site, and blunt dissection was required to expose the nerve (Table 3). There were no statistically significant differences in the degree of perineural adhesion between the control and each experimental group. In particular, *Coptis japonica* (Thunb.) Makino did not cause any severe adhesion.

**Table 3:** Results of the numerical grading system for gross evaluation for each group according to the Peterson classification [15]

	Skin and muscle fascia			Nerve adherence		
	Grade 1	Grade 2	Grade 3	Grade 1	Grade 2	Grade 3
Group 1 (11)	11	0	0	2	6	3
Group 2 (9)	9	0	0	0	6	3
Group 3 (10)	10	0	0	0	10	0
Group 4 (8)	8	0	0	0	6	2
Group 5 (10)	10	0	0	1	6	3
Total (48)	48 (100%)	0	0	3 (6.3%)	34 (70.8%)	11 (22.9%)

Group 1: Control, Group 2: *Astragalus mongholicus* Bunge, Group 3: *Coptis japonica* (Thunb.) Makino, Group 4: *Aconitum carmichaelii* Debeaux, Group 5: *Paeonia lactiflora* Pall

**Fig. 6:**
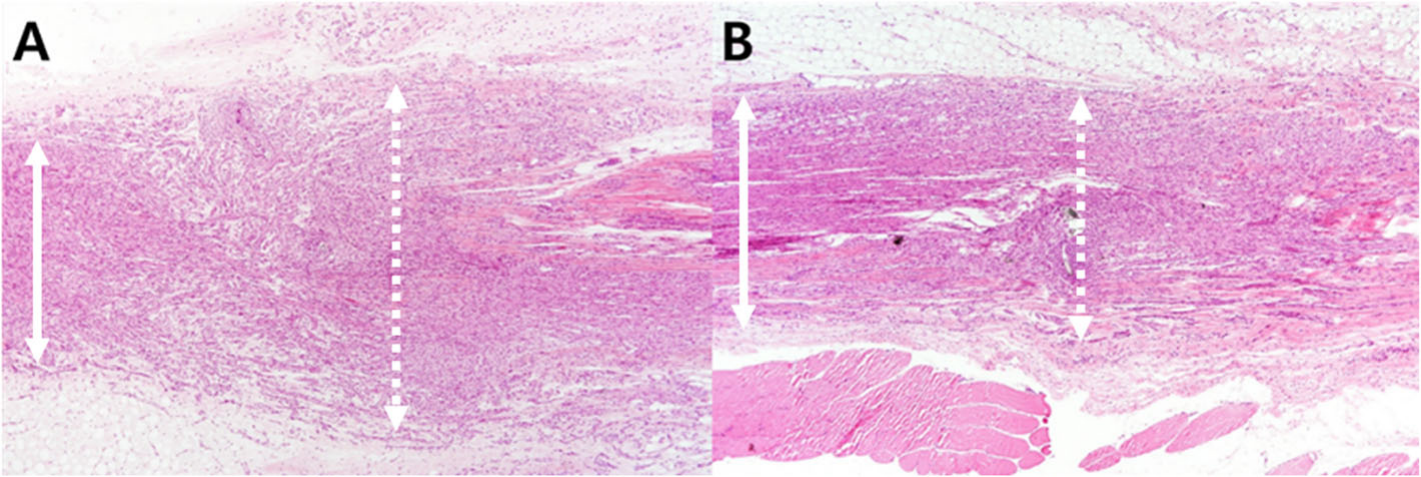
Photomicrographs showing longitudinal sections of the sciatic nerve following neurorrhaphy in the control group (**A**) and in the *Paeonia lactiflora* Pall. group (**B**). The sections were stained with H&E and magnified × 40. The infiltration of inflammatory cell and the parallel appearance of axons were observed in the repaired nerve. The nerve diameter of the repaired nerve (dashed line; middle portion of each photograph) was thicker than the diameter of the proximal nerve (solid line; left side of each photograph), and the measured ratio of the neuroma size was 1.4 in A and 1.0 in B

**Fig. 7:**
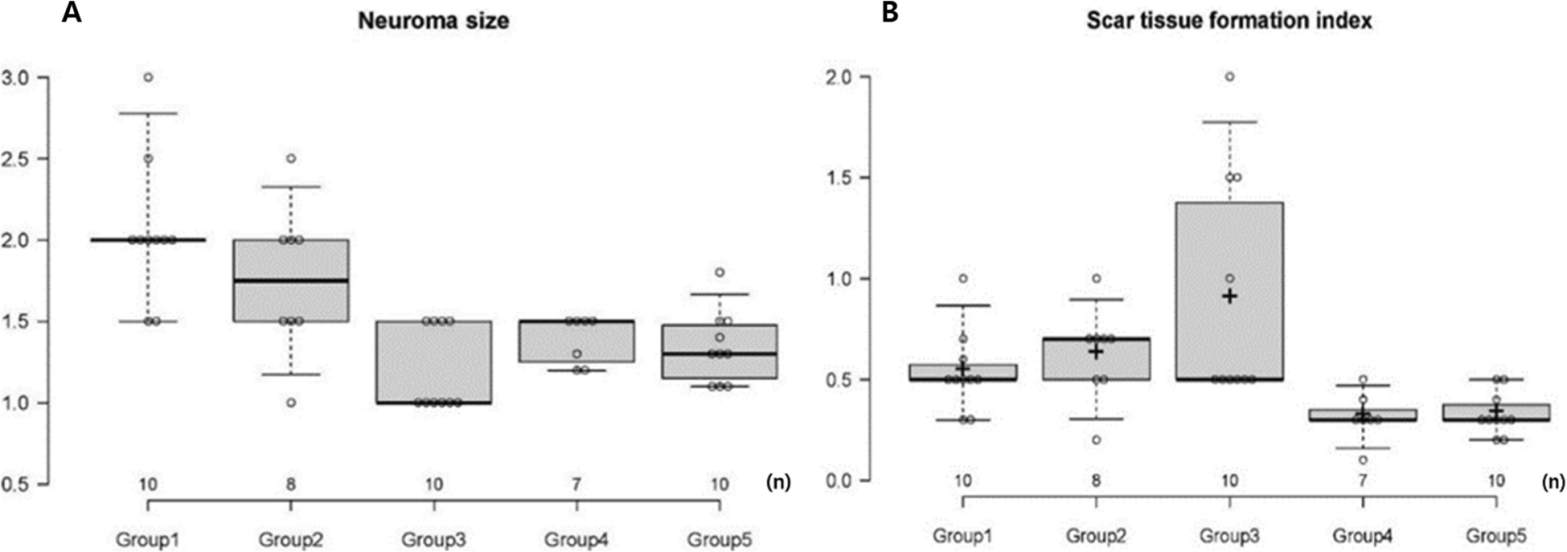
Results of the neuroma size and scar tissue formation index for each group. The neuroma size was smaller in all experimental groups than in the control group and the difference was statistically significant for Groups 3, 4, 5 (*p* = 0.00013, 0.001, 0.00013, respectively). The scar tissue formation index was lower in Groups 4 and 5 than in the control group and the difference was statistically significant for Group 5 (*p* = 0.009). Group 1: Control, Group 2: *Astragalus mongholicus* Bunge, Group 3: *Coptis japonica* (Thunb.) Makino, Group 4: *Aconitum carmichaelii* Debeaux, Group 5: *Paeonia lactiflora* Pall. Neuroma size = ratio of the diameter of the nerve on the site of neurorrhaphy to that on the normal proximal nerve. Scar tissue formation index = ratio of the thickness of the epineural scar tissue to the nerve diameter

Microscopic evaluations were performed on 45 subjects and did not include the five subjects who had died before the re-operation or the three subjects in which anastomosis failure was observed during the morphological evaluation (Fig. 2). The neuroma sizes at the neurorrhaphy site were 1.75, 1.20, 1.39, and 1.34, for the *Astragalus mongholicus* Bunge, *Coptis japonica* (Thunb.) Makino, *Aconitum carmichaelii* Debeaux, and *Paeonia lactiflora* Pall. groups, respectively, all of which were lower than the average of 2.05 of the control group. In the statistical analysis, the *p* values were 0.00013, 0.001, and 0.00013 for the *Coptis japonica* (Thunb.) Makino, *Aconitum carmichaelii* Debeaux, and *Paeonia lactiflora* Pall. groups, respectively, showing significant differences when compared with the control group (Fig. 6, Table 4). The scar tissue formation index was 0.31 in the *Aconitum carmichaelii* Debeaux group and 0.33 in the *Paeonia lactiflora* Pall. group, both of which were lower than the index 0.54 of the control group. In particular, *Paeonia lactiflora* Pall. showed a statistically significant decrease in scar tissue (Fig. 7, Table 4).

**Table 4:** Results of the neuroma size and the scar formation index for each group

Group (n)	Neuroma size Mean (range)	Scar formation index Mean (range)
Group 1 (10)	2.05 (1.5–3.0)	0.54 (0.3–1.0)
Group 2 (8)	1.75 (1.0–2.5)	0.63 (0.2–1.0)
Group 3 (10)	1.20 (1.0–1.5)	0.9 (0.5–2.0)
Group 4 (7)	1.39 (1.2–1.5)	0.31 (0.1–0.5)
Group 5 (10)	1.34 (1.1–1.8)	0.33 (0.2–0.5)

Group 1: Control, Group 2: *Astragalus mongholicus* Bunge, Group 3: *Coptis japonica* (Thunb.) Makino, Group 4: *Aconitum carmichaelii* Debeaux, Group 5: *Paeonia lactiflora* Pall

Neuroma size = ratio of the diameter of the nerve on the site of neurorrhaphy to that on the normal proximal nerve

Scar tissue formation index = ratio of the thickness of the epineural scar tissue to the nerve diameter (Fig. 1)

## Discussion

According to the results of a 40-year clinical follow-up of direct nerve anastomosis performed by Mackinnon et al. [17], only 20–40% of the cases showed very good results with muscle strength at grade 4 and sensory grade 3+ or higher, and almost no complete recovery was observed. Despite numerous studies on the development of microsurgical techniques and nerve regeneration, no method has been discovered that has led to a significant improvement in prognosis, other than the placement of a precise suture.

In axonotmesis, the endoneurium is preserved, which allows the axon an intact pathway to sprout regenerated nerves towards the target organs. However, in the case of neurotmesis, there is no continual neuronal pathway left intact, which means that no regeneration of the nerve can occur without surgery. Lichtman et al. previously reported that the degree to which an axon reached distal motion and sensory target organs was significantly reduced due to scar tissue formation and ineffective axon regeneration in severe nerve injury models [18].

Growth regeneration is also influenced by various neurotrophic factors and neurite-promoting factors. Nerve growth factors have received the most attention in studies on peripheral nerve suture [19]. Specifically, factors influencing nerve regeneration include glial growth factors, fibroblast growth factors, glial cell-derived neurotrophic factors, and neurotrophin 3 [20]. These are known to influence the regeneration of nerve myelin and to improve nerve conduction velocity in the conduit lumen in a sciatic nerve injury model [21, 22]. However, most of the results have been based on animal experiments and have not been clinically applicable [23]. Recent developments in molecular biology have revealed the main molecular biologic pathways of nerve regeneration, and the drugs affecting this pathway have been studied. These drugs include erythropoietin, tacrolimus, acetyl-L-carnitine, and N-acetylcysteine, all of which have been used clinically for a different condition. These have shown effects on potential nerve regeneration; however, the studies performed have been limited to cell cultures and animal experiments and are still in the beginning stages [24].

The effect of *Astragalus mongholicus* Bunge on nerve regeneration has been demonstrated in several previous studies [25–28]. Astragaloside IV, a major active ingredient of *Astragalus mongholicus* Bunge, has been shown to have anti-inflammatory, antioxidant, and anti-apoptotic properties [27]. The effects of nerve regeneration in cerebral ischemia-reperfusion injury have been studied in the central nervous system [28]. In recent years, studies have been conducted on peripheral nerve injury. Zhang et al. performed end-to-end anastomosis in a rat sciatic nerve transection model, which is similar to the model used in this study [28]. Then, they administered astragaloside via intraperitoneal injection. The results indicated that the number and diameter of myelinated nerve fibers were increased. The nerve conduction study also showed increased conduction velocity and amplitude. The molecular biological test also suggested that the mechanism was through an increase in growth-related protein 43 expression. In vitro studies also showed that *Astragali* radix extract promoted nerve growth factor-mediated neurite outgrowth, growth-related-protein 43 expression, and Schwann cell migration [26, 29]. A rat model study of oxaliplatin-induced neuropathy demonstrated the anti-neuropathic effect of *Astragalus mongholicus* Bunge extract, highlighting its potential as a useful natural product for neuropathy [30].

*Coptis japonica* (Thunb.) Makino has been reported to affect axon regeneration [31]. Its major active ingredient, berberine, has been known to help ameliorate damage to the central nervous system caused by cerebral ischemia or Parkinson’s disease [32]. Han et al. [31] reported the regrowth of axons and the increase in thickness of remyelinated axons after intraperitoneal injection of *Coptis japonica* (Thunb.) Makino in a sciatic nerve injury model, thereby indicating the regeneration of peripheral nerves. Another study also showed that *Coptis japonica* (Thunb.) Makino promote nerve growth factor-induced differentiation of neurons [33].

*Aconitum carmichaelii* Debeaux are known to help control neuropathic pain. Several studies have revealed the mechanism underlying this analgesic effect [34–37]. Complementary and alternative medicine is used for analgesic and anti-inflammatory purposes [37], and there have been no reports on its nerve regeneration effect.

*Paeonia lactiflora* Pall. is known to have anti-thrombotic, anti-coagulant, and anti-arteriosclerotic effects. It has been used as a medicine for the treatment of cardiac and liver diseases [38]. Paeoniflorin is the main active substance, and its neuroprotective effects have been reported [39].

*Astragalus mongholicus* Bunge is the most studied herbal drug in terms of peripheral nerve regeneration, and its effects have been demonstrated [25–27, 29, 40]. In this study, treatment with *Astragalus mongholicus* Bunge was correlated with increases in both conduction velocity and amplitude in the nerve conduction study. Treatment with *Coptis japonica* (Thunb.) Makino, increased the conduction velocity the most, and the amplitude was increased compared to the control group. *Aconitum carmichaelii* Debeaux showed the greatest amplitude but decreased conduction velocity. In all experimental groups except the *Aconitum carmichaelii* Debeaux group, the conduction velocity was increased compared to that in the control group, thereby showing the possibility of recovery of nerve function.

In a previous rat study, the proliferation of Schwann cells was observed from approximately 3 weeks, and marked numbers of regenerating fibers were observed at 10–14 weeks [41]. Therefore, in this study, a nerve conduction study was performed at an average of 12 weeks after surgery. The examination time seemed appropriate since the nerve regeneration rate of approximately 1 mm per day has been accepted in the case of general axonotmesis. The nerve conduction study reflects the degree of maturation of regenerated nerves [42], but even when nerve regeneration in a muscle is achieved, the atrophy of the immature muscles remains, and the staggered regeneration of the sensory and muscle nerves during regeneration may have impaired the results of the nerve conduction study [29, 43]. Therefore, a nerve conduction study performed after the muscles have matured, through long-term follow-up or molecular biological or immunohistochemical tests, may be helpful in demonstrating the effect of herbal drugs.

Another factor that adversely affects the outcome of nerve regeneration is scar tissue formation that occurs following nerve regeneration. Intraneural scarring, caused by surgery or hemorrhage, acts as a physical barrier to axon regeneration and interferes with nerve conduction [44]. Extraneural scarring promotes nerve compression or tethering effects, resulting in the contraction of nerve vessels, ischemia, or irreversible damage to the nerves due to strain [45]. There have been various studies to reduce this nerve adhesion, including surgical methods such as wrapping the paraneural areas with flap surgery, fat grafts, and the use of a silicon conduit. As a drug administration method, topical administration of hyaluronic acid has been reported [16]. In a rat model that underwent anastomosis after sciatic nerve transection, which is similar to the model in this study, perineural scar tissue formation was reduced [16]. Although it was reported that drugs did not interfere with the regeneration of the nerve that underwent end-to-end anastomosis [46], there have been few studies on herbal drugs.

According to the results of this study, the control, *Astragalus mongholicus* Bunge, and *Aconitum carmichaelii* Debeaux groups each had one case where the anastomosis failed, while the *Coptis japonica* (Thunb.) Makino and *Paeonia lactiflora* Pall. groups did not have this difficulty. The failed anastomosis may have arisen from a weak degree of anastomosed nerve or anastomotic technique issues rather than the effect of the drugs since the activities of the rats were not limited after anastomosis. Furthermore, drugs administered topically to the surgical site may have side effects such as delayed wound healing due to inflammation and adhesion of surrounding tissues, and excessive perineural scar tissues that are associated with neurological disorders [47]. Moreover, the administered drugs may leak into the muscle and skin sutures and inhibit the healing of suture edges [48]. However, in this study, the experimental and control groups did not show any difference in terms of wound healing or perineural adhesion, thereby indicating that the drugs and administration methods used did not cause foreign body reactions which could have affected wound healing after surgery. Microscopic evaluation revealed that the size of the neuroma was significantly smaller in the *Coptis japonica* (Thunb.) Makino, *Aconitum carmichaelii* Debeaux, and *Paeonia lactiflora* Pall. groups than in the control group. In particular, the size of the perineural scar tissues at the site of the anastomosis was significantly decreased in the *Paeonia lactiflora* Pall. group. These results have not been reported in the existing animal experiments on peripheral nerve regeneration using herbal extracts, thereby revealing the significance of the results.

In this study, the histological analysis of the neuroma and scar tissue formation after treatment with the four herbal extracts showed significant results. However, there were some limitations to our study. The nerve conduction study confirmed the possibility of recovery of neurological function, but molecular biology and immunohistochemical tests were not performed in this study. In addition, the experimental drugs were administered only once at the time of surgery, and the quantity of the extracts that produced the effects was not established. This is a preliminary study to search for herbal medicines with the potential to facilitate nerve regeneration during microsurgery. In future studies, experimental herbal medicine will be quantitatively analyzed, and the effects of the dosage will be evaluated.

## Conclusion

In this study, the size of the neuroma decreased significantly after treatment with *Coptis japonica* (Thunb.) Makino, *Aconitum carmichaelii* Debeaux, and *Paeonia lactiflora* Pall. compared to the control. The *Paeonia lactiflora* Pall. group also showed a significant decrease in scar tissue formation. The peripheral nerve regeneration effect of the herbal extracts was confirmed in this study through the decreased neuroma size and scar tissue formation, both of which have not been previously reported. Based on these findings, further studies on the mechanism of these effects and the development of therapeutic drugs are needed.

## Supplementary Information

**Additional file 1.**

**Additional file 2: Figure S1.** The liquid chromatography-mass spectrometry (LCMS) chromatograph of *Astragalus mongholicus* Bunge extracts.

**Additional file 3: Figure S2.** The liquid chromatography-mass spectrometry (LCMS) chromatograph of *Coptis japonica* (Thunb.) Makino extracts.

**Additional file 4: Figure S3.** The liquid chromatography-mass spectrometry (LCMS) chromatograph of *Aconitum carmichaelii* Debeaux extracts.

**Additional file 5: Figure S4.** The liquid chromatography-mass spectrometry (LCMS) chromatograph of *Paeonia lactiflora* Pall. extracts.

## Data Availability

The datasets used or analyzed during the current study are available from the corresponding author upon reasonable request. All data generated during this study are included in this manuscript.

## Abbreviations

DA: Distal amplitude
IACUC: Institutional Animal Care and Use Committee
NCS: Nerve conduction study
PA: Proximal amplitude
