# Supplementary material for: Effect of herbal extracts on peripheral nerve regeneration after microsurgery of the sciatic nerve in rats
Source: BMC Complement Med Ther. 2021 Jun 4;21:162. doi: 10.1186/s12906-021-03335-w (PMC8178854; doi:10.1186/s12906-021-03335-w)
Supplement: Supplementary file 1 — Additional file 1. [file 12906_2021_3335_MOESM1_ESM.docx]

**Additional file 1. Liquid chromatography-mass spectrometric method**

## Materials

## Solvent: 3DW (Aldrich), Acetonitrile (B&J, Merck), Methanol (B&J, Merck)

## Reagent: Formic acid (Aldrich), Ammonium formate (Aldrich)

## Instrument condition -1

## LC Chromatography: Ultimate 3000 RS system (Thermo fisher scientific Inc.)

## Mass spectrometry: LTQ XL (Thermo fisher scientific Inc.)

## Column: U-VDSpher Pur C18-E, 1.8um, 50 x 2.0mm (VDS optilab)

## Solvent: A: 3DW (0.1% Formic acid), B: Acetonitrile (0.1% Formic acid), C: MeOH (0.1% Formic acid), D: Acetonitirle/MeoH(7/3, 0.1% Formic acid)

## Elution condition: Isocratic

## Flow rate: 0.1, 0.2, 0.25, 0.3 ml/min

## Injection volume: 2, 5 uL

## Instrument condition -2

## Scan range: *m/z* 100~2000

## Detection ion mode: Positive ion, [M+H, Na]^+^, Negative ion, [M-H]^-^

## Mass exp.: LTQ exp. Capillary temperature: 300C, Source voltage: 3.5kV(Positive), 2.7kV(Negative), Sheath gas flow: 42

## Software: Xcalibur 2.0, 4.0
