# Supplementary figures and images for: Effect of herbal extracts on peripheral nerve regeneration after microsurgery of the sciatic nerve in rats
Source: BMC Complement Med Ther. 2021 Jun 4;21:162. doi: 10.1186/s12906-021-03335-w (PMC8178854; doi:10.1186/s12906-021-03335-w)

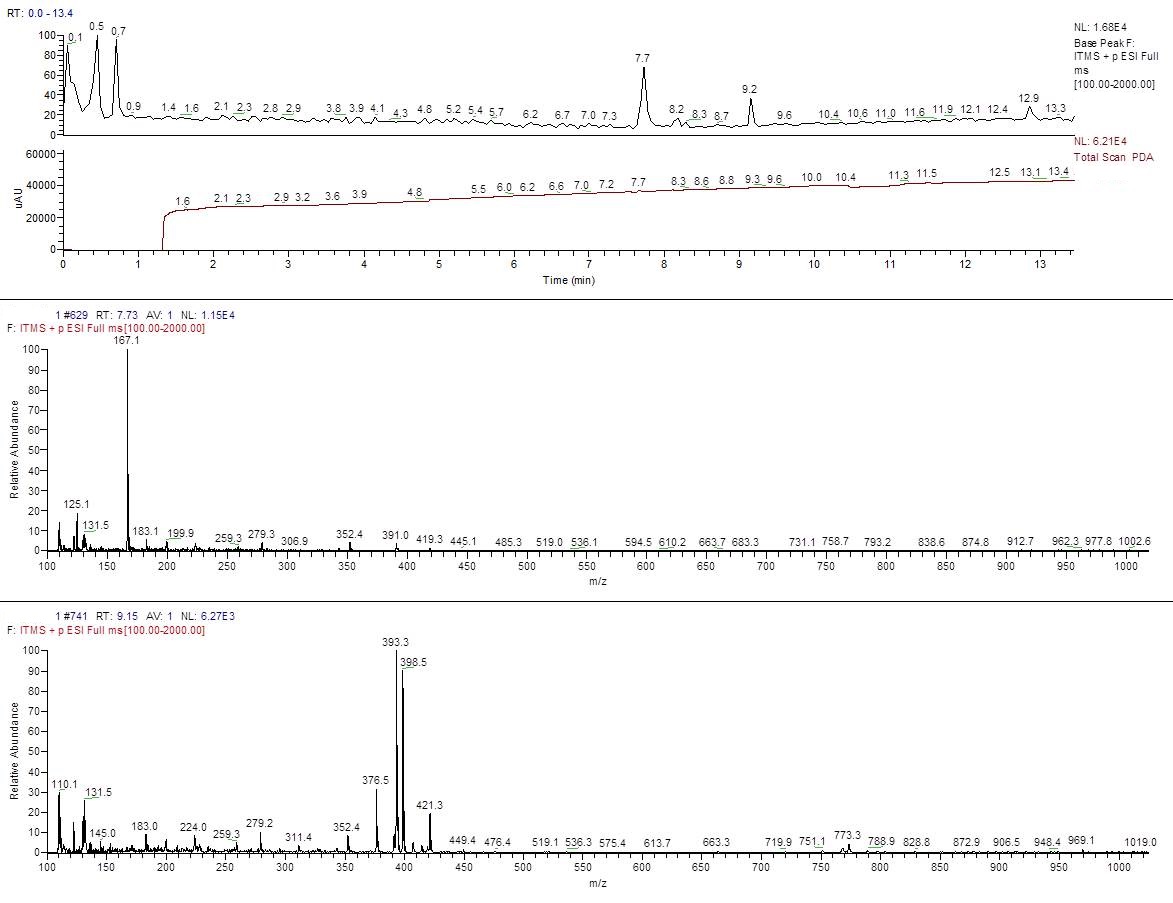

Supplement: Supplementary file 2 — Additional file 2: Figure S1. The liquid chromatography-mass spectrometry (LCMS) chromatograph of Astragalus mongholicus Bunge extracts. [file 12906_2021_3335_MOESM2_ESM.jpg]

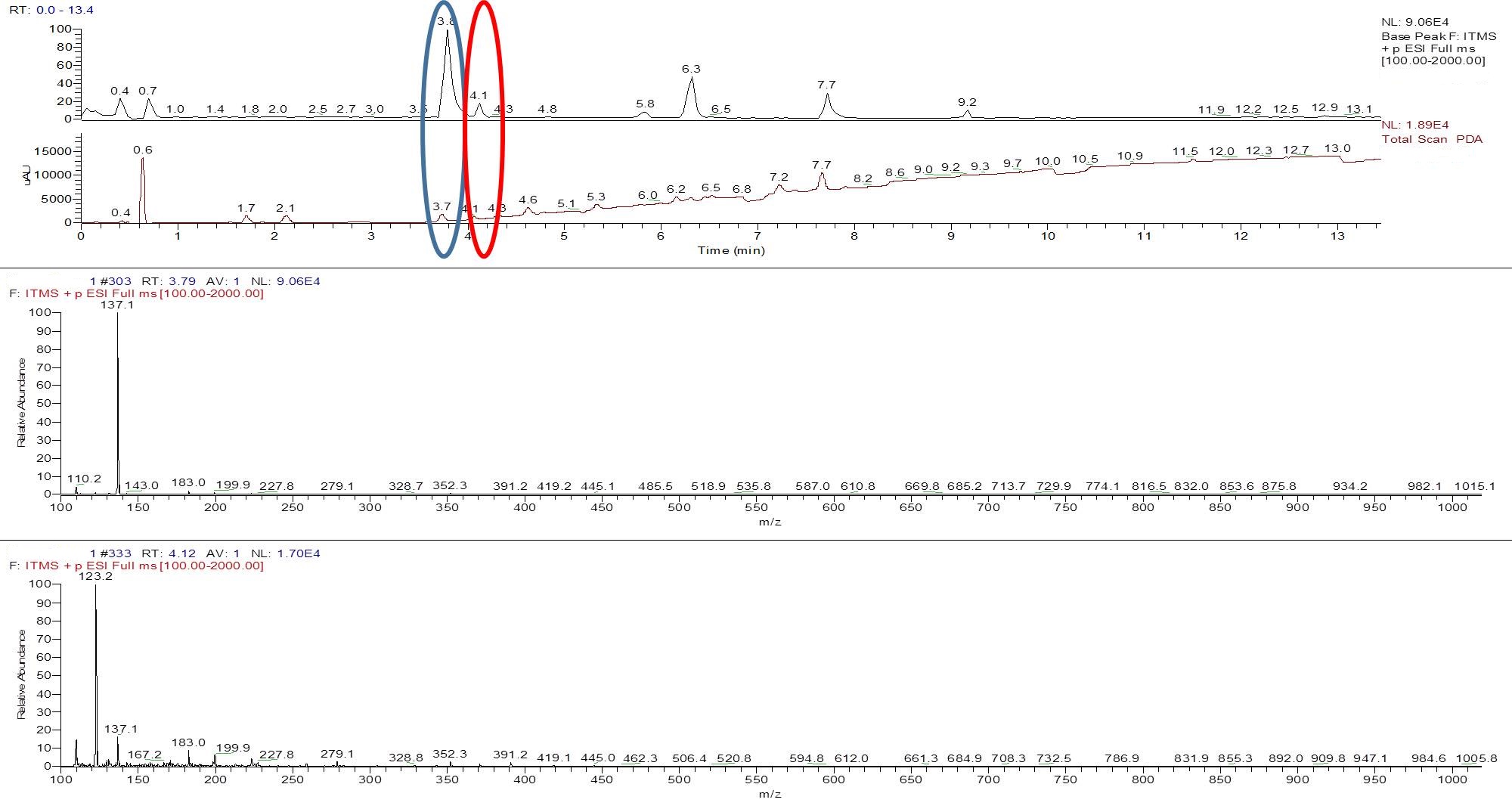

Supplement: Supplementary file 3 — Additional file 3: Figure S2. The liquid chromatography-mass spectrometry (LCMS) chromatograph of Coptis japonica (Thunb.) Makino extracts. [file 12906_2021_3335_MOESM3_ESM.zip › Supplementary figure 2_A_final_ChoR2.jpg]

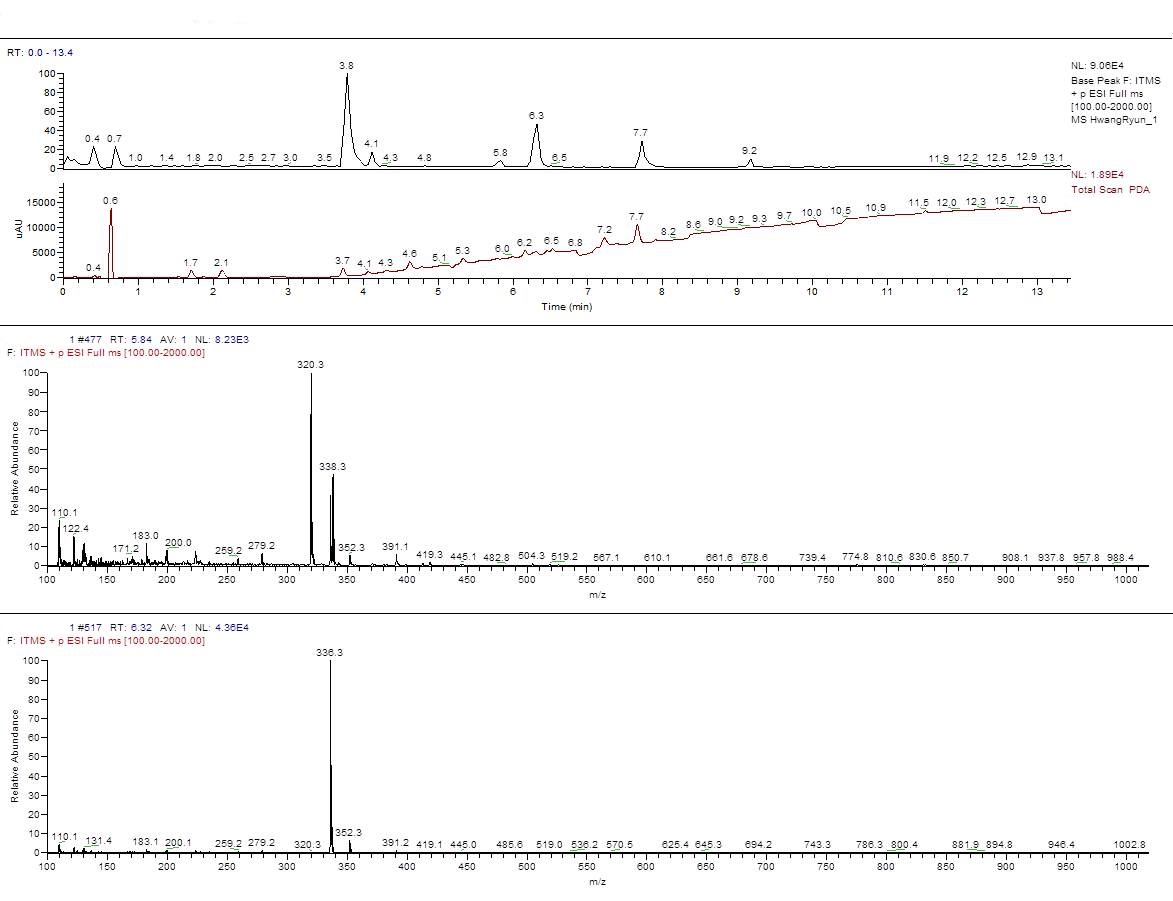

Supplement: Supplementary file 3 — Additional file 3: Figure S2. The liquid chromatography-mass spectrometry (LCMS) chromatograph of Coptis japonica (Thunb.) Makino extracts. [file 12906_2021_3335_MOESM3_ESM.zip › Supplementary figure 2_B_final_ChoR2.jpg]

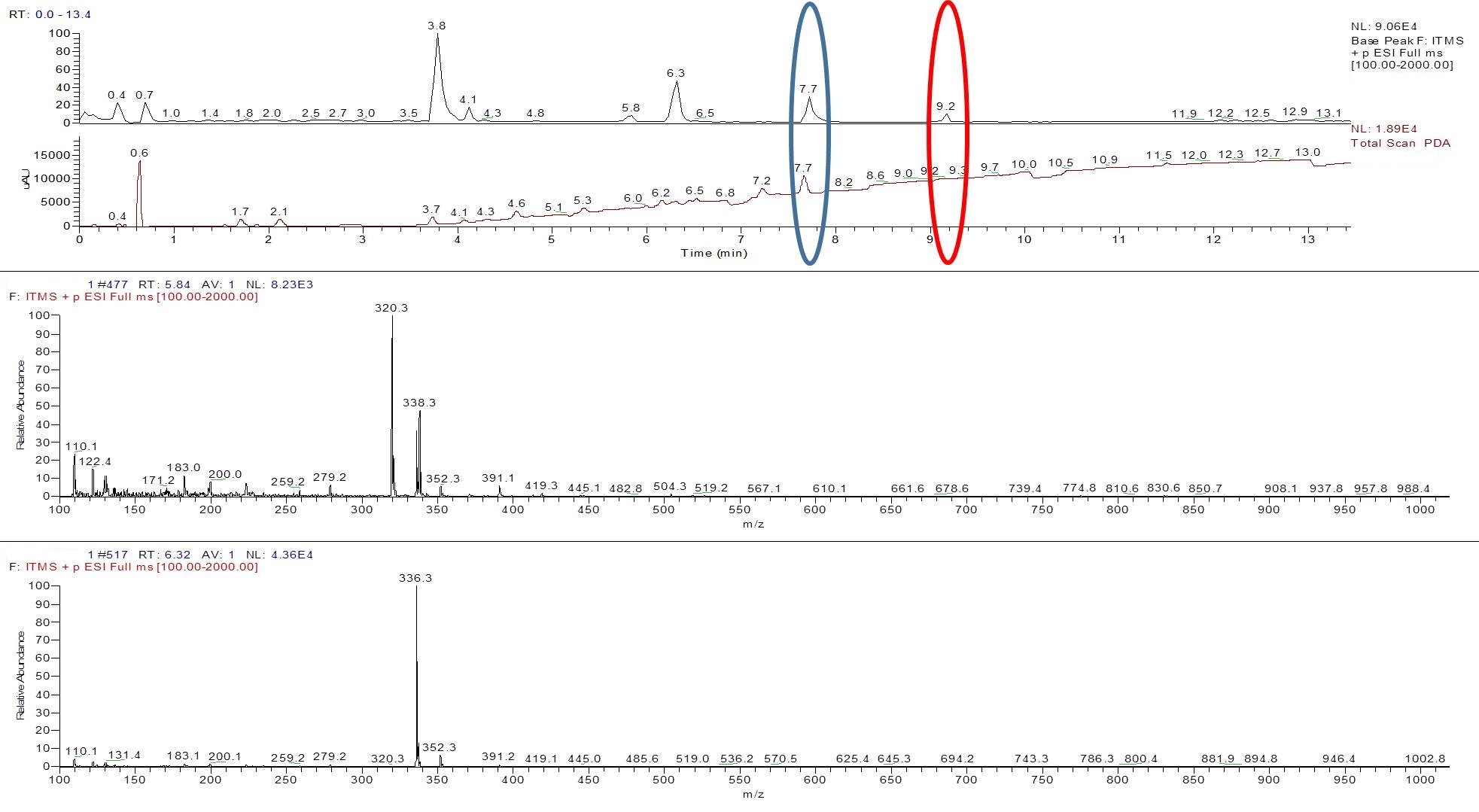

Supplement: Supplementary file 3 — Additional file 3: Figure S2. The liquid chromatography-mass spectrometry (LCMS) chromatograph of Coptis japonica (Thunb.) Makino extracts. [file 12906_2021_3335_MOESM3_ESM.zip › Supplementary figure 2_C_final_ChoR2.jpg]

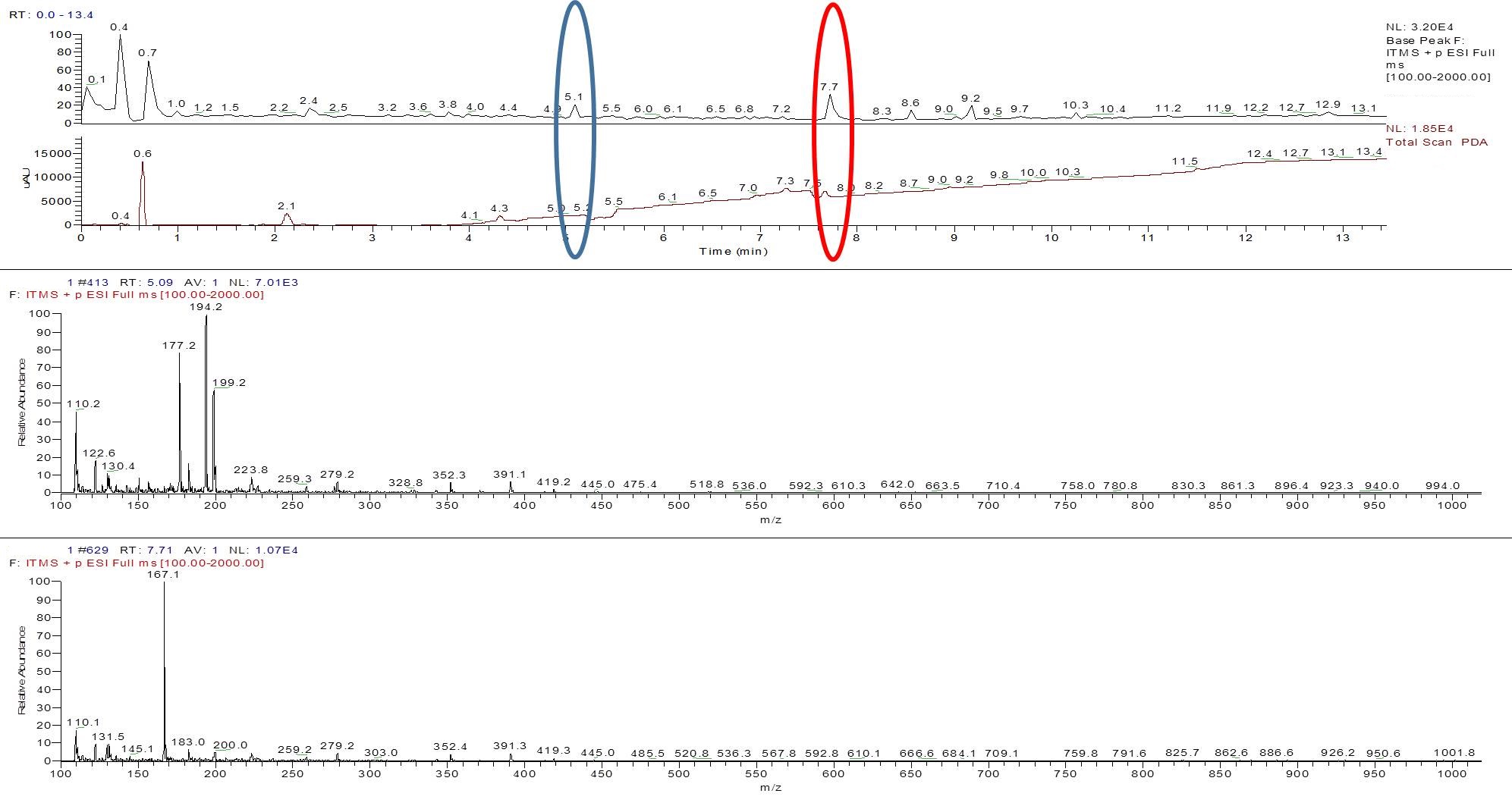

Supplement: Supplementary file 4 — Additional file 4: Figure S3. The liquid chromatography-mass spectrometry (LCMS) chromatograph of Aconitum carmichaelii Debeaux extracts. [file 12906_2021_3335_MOESM4_ESM.zip › Supplementary figure 3_A_final_ChoR2.jpg]

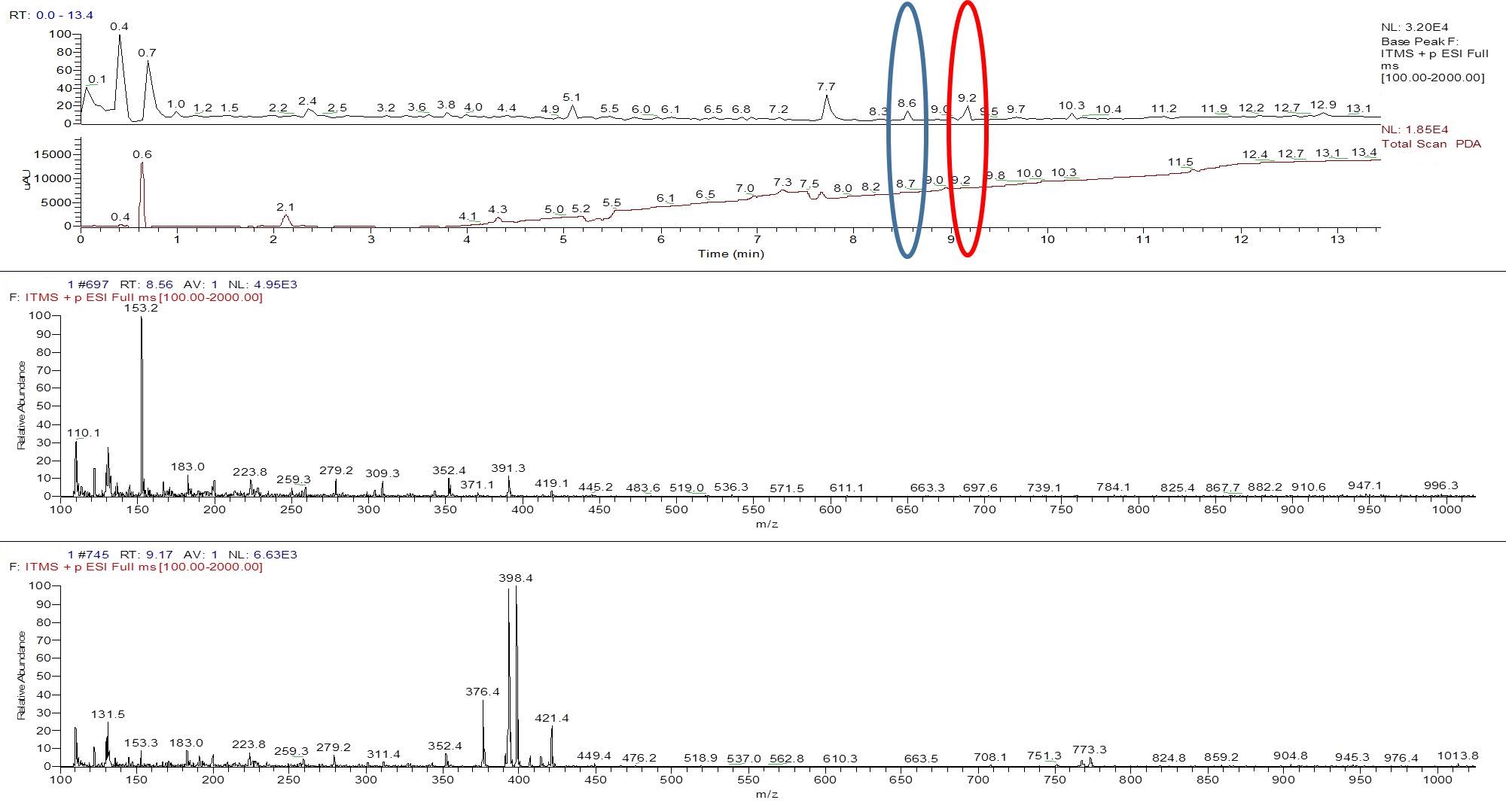

Supplement: Supplementary file 4 — Additional file 4: Figure S3. The liquid chromatography-mass spectrometry (LCMS) chromatograph of Aconitum carmichaelii Debeaux extracts. [file 12906_2021_3335_MOESM4_ESM.zip › Supplementary figure 3_B_final_ChoR2.jpg]

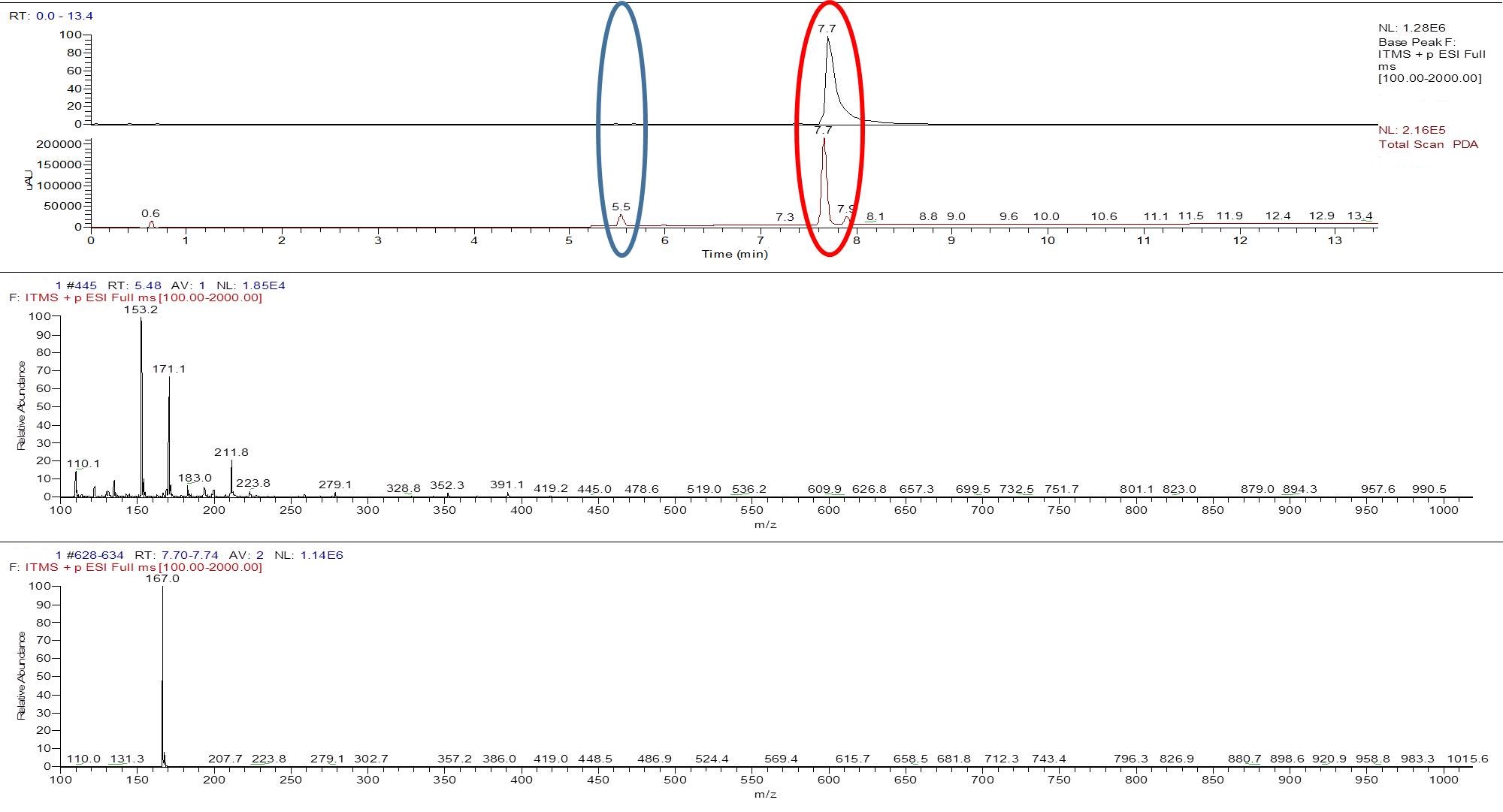

Supplement: Supplementary file 5 — Additional file 5: Figure S4. The liquid chromatography-mass spectrometry (LCMS) chromatograph of Paeonia lactiflora Pall. extracts. [file 12906_2021_3335_MOESM5_ESM.jpg]
